# Transforming Growth Factor ****β****1 Genotypes in Relation to TGF****β****1, Interleukin-8, and Tumor Necrosis Factor Alpha in Induced Sputum and Blood in Cystic Fibrosis

**DOI:** 10.1155/2013/913135

**Published:** 2013-08-26

**Authors:** O. Eickmeier, L. v. d. Boom, F. Schreiner, M. J. Lentze, D. NGampolo, R. Schubert, S. Zielen, S. Schmitt-Grohé

**Affiliations:** ^1^Department of Pediatric Pulmonary, Goethe University Hospital, Theodor-Stern-Kai 7, 60590 Frankfurt, Germany; ^2^Department of Pediatrics, University Hospital, Adenauerallee 119, 53113 Bonn, Germany

## Abstract

*Background*. High-producer TGF**β**1 genotypes are associated with severe lung disease in cystic fibrosis (CF), but studies combining IL-8, TNF**α**-, and TGF**β**1(+genotype) levels and their impact on CF lung disease are scarce. *Aim*. Assessing the relationship between TGF**β**1, IL-8, and TNF-**α** and lung disease in CF in an exacerbation-free interval. *Methods*. Twenty four patients delta F508 homozygous (median age 20.5 y, Shwachman score 75, FEV1(%) 83) and 8 controls (median age 27.5 y) were examined. TGF**β**1 was assessed in serum and induced sputum (IS) by ELISA, for IL-8 and TNF-**α** by chemiluminescence in IS and whole blood. Genotyping was performed for TGF**β**1 C−509T and T+869C utilizing RFLP. *Results*. TGF**β**1 in IS (CF/controls median 76.5/59.1 pg/mL, *P* < 0.074) was higher in CF. There was a negative correlation between TGF**β**1 in serum and lung function (LF) (FEV1 (*r* = −0.488, *P* = 0.025), MEF 25 (*r* = −0.425, *P* = 0.055), and VC (*r* = −0.572, *P* = 0.007)). Genotypes had no impact on TGF**β**1 in IS, serum, and LF. In IS TGF**β**1 correlated with IL-8 (*r* = 0.593, *P* < 0.007) and TNF-**α** (*r* = 0.536, *P* < 0.018) in patients colonized by bacteria with flagellin. *Conclusion*. TGF**β**1 in serum not in IS correlates with LF. In patients colonized by bacteria with flagellin, TGF**β**1 correlates with IL-8 and TNF-**α** in IS.

## 1. Introduction

It is well known that a higher TGF*β*1 production and a high-producer TGF*β*1 genotype are associated with the development of increased lung fibrosis [1]. Arkwright et al. [1] provided as the first evidence that individuals with the TGF*β*1 +869TT genotype developed a more rapid decline in FEV1 compared to patients carrying one or two TGF*β*1 +869C alleles in a cohort of 171 CFTR 508del homozygous patients from the UK. Though they could not confirm this association in 261 CF patients with delta F508 homozygous or compound mutations from Classes I, II and III in the CFTR gene [2]. The association of TGF*β*1 codon 10 CC genotype and the TT genotype of −509 with more severe lung disease in delta F 508 homozygous cystic fibrosis patients was shown by Drumm and coworkers [3] in a large trial involving 808 CFTR F508 del homozygous patients. In contrast to the findings of Arkwright, they showed a significant association of the TGF*β*1 +869CC and not +869TT genotype with more severe lung disease. A cohort of 329 French and German CF patients (out of them 171 F508del homozygous) were examined by Corvol et al. [4]. A less pronounced rate of decline in forced expiratory volume in 1 sec (FEV1) and forced vital capacity (FVC) in patients heterozygous for TGF*β*1 +869 (+869CT) was observed, when compared to patients carrying either TGF*β*1 +869TT or +869CC genotypes.

TGF*β*1 is secreted by endothelial, hematopoietic, and connective tissue cells [5]. Major effects include inhibition of epithelial proliferation, induction of expression of genes encoding components of the extracellular matrix promoting fibrosis, and inhibition of expression of metalloprotease genes.

TGF*β*1 is an important effector molecule in the development of pulmonary edema after acute lung injury [6]. Blocking TGF*β*1 also prevents pulmonary edema in response to intratracheal endotoxin [7]. It directly increases the permeability of pulmonary endothelial monolayers and also increases the permeability of alveolar epithelial monolayers [6]. 

So far, few studies have measured TGF*β*1 levels in cystic fibrosis patients. One study measured TGF*β*1 in plasma of CF patients with a forced expiratory volume in 1 second (FEV1) <40% and found significantly higher values than in controls [8]. A recent study [9] has evaluated TGF*β*1 in 11 samples of bronchoalveolar lavage samples in infants and young children and found a direct correlation with functional residual capacity (FRC). 

Another study evaluated TGF*β*1 in BAL in older children, with a pulmonary exacerbation [10]. TGF*β*1 was elevated compared to controls and increase was associated with neutrophilic inflammation, diminished lung function, and recent hospitalization. In a second study [11] this group obtained TGF*β*1 in BAL as well as in serum and plasma in children (pre- and postintravenous antibiotic therapy) hospitalized for pulmonary exacerbation. Plasma TGF*β*1 was inversely associated with diminished lung function after therapy. Both studies describe short-time incidents. 

To our knowledge there is no data comparing TGF*β*1 levels in serum and in induced sputum and relating it to clinical data in an exacerbation-free interval which should reflect the long-term effect. We prefer that induced sputum as bronchoalveolar lavage is an invasive procedure and potentially risky and harmful to the patient.

There is scarce evidence that high TGF*β*1 levels do have an impact on cystic fibrosis lung disease. So far it was shown that there is an association of such genotypes (that postulate high producer phenotypes) with severe lung disease in CF.

Recent research focused on the impact of bacterial flagellin on the expression of TGF*β*1 and interleukin-8 [12] via mitogen-activated protein kinases (MAPK) in normal bronchial epithelial cells. So we were interested in Interleukin-8 (IL-8) and tumor necrosis factor alpha (TNF-alpha) as well. To our knowledge, there is so far no data comparing TGF*β*1 and cytokines like IL-8 and TNF-alpha in induced sputum and blood in CF.

As TGF*β*1 not only promotes fibrosis but also increases alveolar endothelial permeability, this might promote transfer of hematopoietic cells and TGF*β*1 from blood into the lung. So we were interested in the relationship of TGF*β*1 and leukocytes measured in induced sputum as well as in blood. To adjust for genetic heterogenicity in the CFTR gene only, individuals homozygous for delta F 508 were enrolled. As TGF*β*1 inhibits CFTR biogenesis and prevents functional rescue of del F 508 in human bronchial epithelial cells [13], it seems even more important to examine individuals homogenous for del F 508.

Patients were examined in an exacerbation-free interval to exclude the stimulatory effects of ongoing acute infections. 

The aim of the study was to provide evidence that TGF*β*1 genotypes (+869T->C, −509C->T) as modifiers of CF lung disease correlate with *ex vivo* TGF*β*1 assessed in serum and sputum in delta F 508 homozygous CF patients. Moreover, we were interested in the interaction of TGF*β*1 and the levels of other proinflammatory cytokines in blood and sputum.

The findings of this study provide evidence that lung function correlates with TGF*β*1 in serum but not in induced sputum. Genotypes in this small cohort did not have an impact on TGF*β*1 in serum, sputum or lung function. There was a significant correlation between TGF*β*1 in sputum and IL-8 as well as TNF-alpha in patients colonized by bacteria with flagellin.

## 2. Methods

### 2.1. Subjects

Patients with the delta F 508 (homozygous) mutation were recruited from the Cystic Fibrosis Outpatient Clinic of the Children's Hospital Medical Center at the University of Bonn and the Department of Pediatric Pulmonary of the University of Frankfurt. Healthy individuals served as controls. Exclusion criteria were clinical or laboratory signs (CRP > 20 mg/L) of an exacerbation, treatment with systemic steroids 14 days preceding this trial, or participation in another study within the past 30 days. All subjects performed spirometry, gave a blood sample, and induced sputum. The protocol was approved by the ethics committee of the Universities of Bonn and Frankfurt, respectively. Informed consent was obtained from all patients, respective parents.

### 2.2. Spirometry

Lung function tests (vital capacity (VC), forced expiratory volume in one second (FEV1), maximum expiratory flow at 25% of forced vital capacity (MEF25)) were performed using a Master Screen Body, Fa. Viasys, Wuerzburg, Germany. To accurately assess the individual lung function, the median of three lung function tests (LFT No. 1–3) was used (No. 1 LFT app. 3 months before the cytokine measures were taken, No. 2 at the time the sputum and blood sample was provided, and No. 3 LFT app. 3 months after that).

### 2.3. Cytokine Assessment

Blood (9 mL) was collected in endotoxin-free collection tubes (EDTA, SARSTEDT Monovette, Nuembrecht, Germany). TGF*β*1 was assessed in serum as well as in induced sputum (IS) by an ELISA kit (R&-D Systems, Wiesbaden, Germany) according to the manufacturer's manual. 

According to the manufacturer, this assay has a high intra-assay precision for TGF*β*1 (coefficient of variation (CV) of 1.9–2.9%), interassay precision (CV of 6.4–9.3%), and a sensitivity (mean) of 4.61 pg/mL). Concerning specificity as the assay recognizes both natural and recombinant TGF*β*1, cross-reactivity was tested, and no significant cross-reactivity or interference was observed.

Interleukin-8 (IL-8) and TNF alpha in whole blood were measured as previously described [14, 15]. In sputum (after processing following the subsequently mentioned protocol) IL-8 and TNF alpha were assessed by chemiluminescence (Immulite, Siemens Healthcare Diagnostics, Eschborn, Germany, formerly DPC Biermann).

### 2.4. Transforming Growth Factor *β* Genotype

DNA was extracted from EDTA blood samples using the QIAamp Kit (Qiagen, Germany). Genotypes for polymorphisms +869T/C and −509C/T were determined by standard PCR and restriction fragment length polymorphism (RFLP) after digestion with enzymes MspA1I (+869T/C) and Bsu36I (−509C/T), respectively. Primer sequences and reaction conditions are available on request. 

### 2.5. Sputum Processing, Cell Counting, and Cell Differentiation

The original protocol for sputum processing had been developed by Holz et al. [16] for preparation of induced sputum in asthma patients. Sputum of CF patients is viscous, often purulent and firm, and could not be processed by this method. So the protocol was modified to be suitable for CF sputum.

Inhalation of antibiotics was performed 8 hours prior to the sputum induction. In order to minimize bronchial constriction, patients inhaled 200 *μ*g of salbutamol 10 minutes prior to sputum induction and then 3% saline solution for 10 to 12 minutes. The patients then expectorated sputum in a sputum tube. This was immediately put on ice and processed in the laboratory.

The sputum was weighed and transferred into a conical tube. The sample was diluted 1 : 3 with dithiothreitol 0.1% (DTT, Sigma Germany), vortexed, and incubated for 15 minutes at 38°C with constant agitation. Thereafter, it was diluted 1 : 5 with phosphate buffered saline (PBS, Gibco, Invitrogen Corp., UK) and vortexed. Some undissolved sputum residues in the specimen were filtered with a cell strainer, placed into a new, weighed conical tube, and then centrifuged with the strainer (Falcon, USA) for 10 minutes at 350 g. After this, there was sputum residue in the cell strainer, as well as a supernatant and a cell pellet in the tube. The tube was again weighed without the cell strainer in order to calculate the amount of sputum in the tube as well as sputum residue in the cell strainer. The supernatant was distributed into 6 Eppendorf tubes and frozen at −80°C for cytokine analysis.

The cell pellet was resuspended with 500 *μ*L PBS/bovine serum albumin (BSA, Serva) 2%. An aliquot was diluted 10 : 1 with Trypan blue 0.4% (Sigma Aldrich, Germany) and a cell count was created by using a hemocytometer, and the amount of viable and dead cells (also using Trypan blue) was subsequently calculated.

### 2.6. Statistical Analysis

We compared outcomes for two groups using the Mann-Whitney *U* test for unpaired samples and the Wilcoxon test for paired samples. The correlations between quantitative data were estimated using Spearman correlation coefficient. All calculations were done by SPSS (Version 21.0).

## 3. Results

### 3.1. Patient Characteristics

Twenty-four patients (delta F 508 homozygous) (14 male and 10 female subjects) and eight controls (3 male and 5 female subjects) were recruited. Median age was 20.5 years (range 6–44 years) in patients and 27.5 years (range 25–38 years) in controls. Transforming growth factor *β*1 (TGF*β*1) into sputum was available from all patients and controls, and TGF*β*1 in serum was measured in 22 CF patients. Leukocyte counts in sputum were available in 23 patients and 8 controls. Blood leukocytes were counted in 23 patients and 4 controls. Twenty three patients were able to perform lung function. Nine patients had microbiologic evidence of *Pseudomonas aeruginosa *colonization. Eleven patients were colonized with *S. aureus*, 3 of them as well with *Pseudomonas aeruginosa.* Stenotrophomonas was detected in 5 patients. *Serratia* in 1 patient, who was colonized with *Pseudomonas aeruginosa *as well. *Candida* was found in 8 patients and 5 of them were colonized with *Pseudomonas aeruginosa* as well. There was microbiologic evidence of *Aspergillus fumigatus* in 6 patients; one was positive for *Pseudomonas aeruginosa* as well. Details are shown in Table 1. 

### 3.2. Transforming Growth Factor *β* in Serum and Induced Sputum

**Figure 1:**
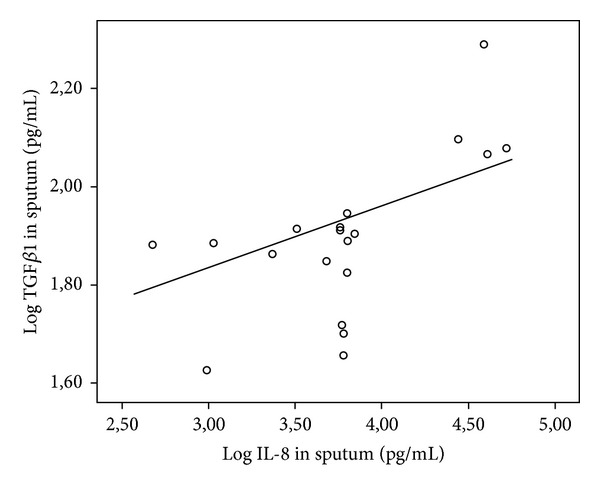
Log 10 TGF*β*1 (pg/mL) in induced sputum in CF patients colonized by either *P. aeruginosa, S. aureus, or S. maltophilia* plotted as a function of Log 10 IL-8 with linear regression line (*r* = 0.593, *P* < 0.007).

**Figure 2:**
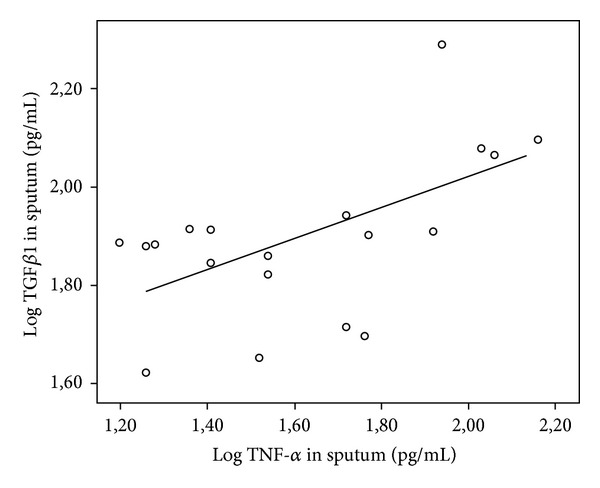
Log 10 TGF*β*1 (pg/mL) in induced sputum in CF patients colonized by either *P. aeruginosa, S. aureus, or S. maltophilia* plotted as a function of Log 10 TNF-alpha with linear regression line (*r* = 0.536, *P* < 0.018).

There was a trend for a significant difference for TGF*β*1 in induced sputum between patients and controls (median 76.5/59.1 pg/mL, mean 79.53/57.85 pg/mL, range 42.2–195/36.1–70.6 pg/mL; *P* < 0.074). In CF patients TGF*β*1 was significantly lower in induced sputum (IS) than in serum (median IS/WB 76.5/35.1 × 10^3^, mean 79.53/34.29 × 10^3^, range 42.2–195/(9.8–53.7) × 10^3^ pg/mL; *P* < 0.0001) (see Table 2). For TGF*β*1 in serum and IL-8 in whole blood there was no correlation. This was also true for TGF*β*1 in serum and TNF alpha in whole blood. But for TGF*β*1, there was a significant correlation with IL-8 (*r* = 0.549, *P* < 0.0007) and TNF alpha (*r* = 0.491, *P* < 0.015) in induced sputum. Analyzing the correlation by carrier status of bacteria with flagellin (*P. aeruginosa, S. aureus, or S. maltophilia*), the data are more distinctive. For those who were negative, there was no correlation, but for those who had microbiological evidence of at least one bacteria the correlation was positive (TGF*β*1/IL-8 *r* = 0.593, *P* < 0.007; TGF*β*1/TNF alpha *r* = 0.536, *P* < 0.018) (see Figures 1 and 2).

### 3.3. Interleukin-8 in Whole Blood and Induced Sputum

For IL-8 in whole blood, there was a significant difference between patients and controls (median 12/5 pg/mL, mean 46.5/10.8 pg/mL, and range 5–357/5–51.4 pg/mL; *P* < 0.008). There was a significant difference for IL-8 in induced sputum between patients, and controls (median 5791/28.3 pg/mL, mean 9901.7/52.5 pg/mL, and range 52847–33.2/148–5 pg/mL; *P* < 0.0001). In CF patients IL-8 was significantly higher in induced sputum (IS) than in whole blood (median IS/WB 5791/12, mean 9901.7/46.5, and range 52847–33.2/5–357 pg/mL; *P* < 0.0001) (Table 3). 

### 3.4. Tumor Necrosis Factor Alpha in Whole Blood and Induced Sputum

For TNF-alpha in whole blood, there was no significant difference between patients and controls (median 11.6/17 pg/mL, mean 64.91/17.2 pg/mL, and range 7.7–1000/8.4–37.3 pg/mL). There was a significant difference for TNF-alpha in induced sputum between patients and controls (median 29.6/16.8 pg/mL, mean 45.8/16.1 pg/mL, and range 13.6–146/13.4–17.8 pg/mL; *P* < 0.0001). In CF patients TNF-alpha was significantly higher in induced sputum (IS) than in whole blood (median IS/WB 29.6/11.6, mean 45.8/64.9, and range 13.6–146/7.7–1000; *P* < 0.024) (Table 3).

### 3.5. Leukocyte Counts

For leukocytes in induced sputum, there was a significant difference between patients and controls (median 520/60, mean 1253/81, and range (2–8480)/(10–240)/*μ*L; *P* < 0.036). There was no significant difference between leukocytes in EDTA blood (median 6600/6800, mean 7353/6763, and range 4200–16290/6000–7450/*μ*L) (Table 2).

### 3.6. Transforming Growth Factor *β*1 and Lung Function

**Figure 3:**
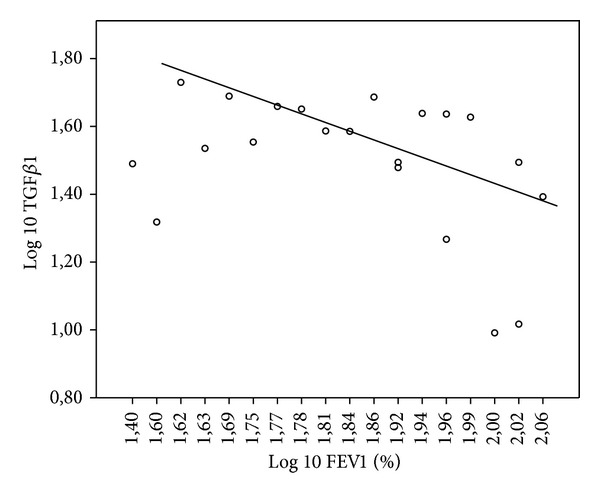
Log 10 TGF*β*1 (pg/mL) in serum in CF patients plotted as a function of Log FEV1 (%) with linear regression line (*r* = −0.488, *P* < 0.025).

**Figure 4:**
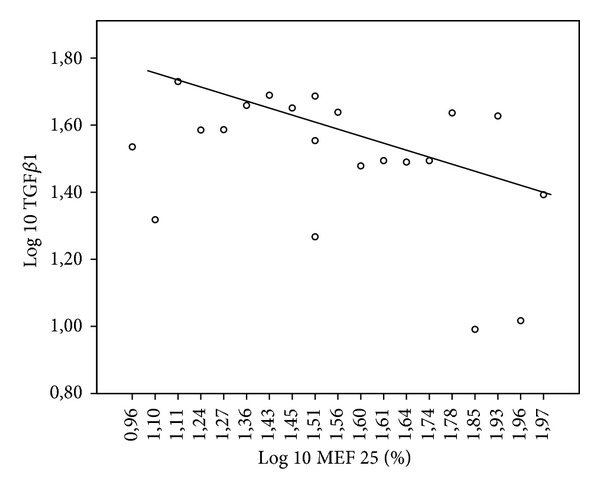
Log 10 TGF*β*1 (pg/mL) in serum in CF patients plotted as a function of Log 10 MEF 25 (%) with linear regression line (*r* = −0.425, *P* < 0.055).

**Figure 5:**
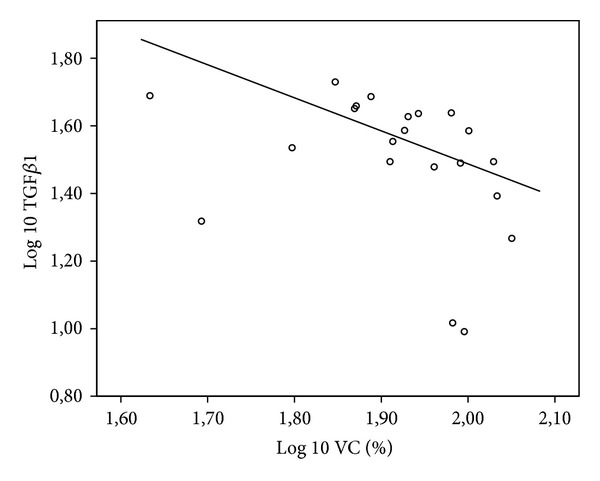
Log 10 TGF*β*1 (pg/mL) in serum in CF patients plotted as a function of Log VC (%) with linear regression line (*r* = −0.572, *P* < 0.007).

As younger children have smaller lung volume, lung function data is only analyzed as percent predicted. There was no correlation between TGF*β*1 in induced sputum and lung function. But there was a negative correlation between TGF*β*1 in serum and FEV1 (*r* = −0.488, *P* = 0.025) (Figure 3). Moreover, there was a trend for a significant correlation between TGF*β*1 in serum and MEF 25 (*r* = −0.425, *P* = 0.055) (Figure 4) and a significant negative correlation between TGF*β*1 in serum and VC (*r* = −0.572, *P* = 0.007) (Figure 5).

### 3.7. Interleukin-8 and Tumor Necrosis Factor Alpha and Lung Function

For IL-8 in induced sputum as well as whole blood, there was no correlation with lung function and between IL-8 in induced sputum and whole blood. But there was a significant negative correlation (*r* = −0.508, *P* < 0.013) for TNF-alpha in whole blood for MEF 25 as well as for FEV1 (*r* = −0.447, *P* < 0.033). For VC, there was no correlation with IL-8 as well as for lung function and tumor necrosis factor alpha in induced sputum.

### 3.8. Transforming Growth Factor *β*1 Genotypes

All subjects were genotyped for TGF-*β*1−509C/T andcodon 10 (T+869C) polymorphism via PCR-RFLP, though this study was not sufficiently powered to evaluate for significant associations between TGF*β*1 genotype and TGF*β*1 serum levels. For C−509T subjects with one T (mutant = CT + TT) polymorphism were combined and for codon 10 we combined subjects with one C polymorphism (mutant = CT + CC).

#### 3.8.1. Patient Characteristics and Influence on TGF*β*1 and Leukocytes in Induced Sputum and Blood

This paragraph refers to the 23 patients who were able to perform lung function. TGF*β*1 mutants (C−509T mutant/wildtype *n* = 9/14 (39%/61%)), respectively, and T+869C mutant (CT + CC)/wildtype (TT) (9/14 (39/61%) were equally distributed among patients. Patient characteristics did not show any significant differences with the exception of the sex distribution (Tables 4 and 5). Genotypes had no influence on levels of TGF*β*1 in induced sputum (C−509T mutant/wildtype median 80.2/71.8 pg/mL; T+869C mutant/wildtype median 80.2/71.8 pg/mL, n.s) and serum (C−509T mutant/wildtype median 35.8 × 10^3^/34.9 × 10^3^ pg/mL, n.s; T+869C mutant/wildtype median 35.8 × 10^3^/34.9 × 10^3^ pg/mL, n.s) on leukocytes in induced sputum (C−509T mutant/wildtype median 651/495/*μ*L, n.s; T+869C mutant/wildtype median 651/495/*μ*L, n.s) and in EDTA blood (C−509T mutant/wildtype median 6325/6990 pg/mL, n.s; T+869C mutant/wildtype median 6325/6990/*μ*L, n.s) (Tables 6 and 7), lung function resp FEV1(%) (C−509T mutant/wildtype median 68.6/86.7%, n.s; T+869C mutant/wildtype median 68.6/86.7%, n.s) or BMI (C−509T mutant/wildtype median 21.9/19.2 pg/mL, n.s; T+869C mutant/wildtype median 21.9/19.2, n.s).

## 4. Discussion

We were able to show that TGF*β*1 in serum correlates negatively with lung function in cystic fibrosis. Moreover, we provided evidence that TGF*β*1 in induced sputum is significantly lower than in serum in CF patients and that there is a trend for significantly higher TGF*β*1 levels in CF patients than in healthy controls for induced sputum in an exacerbation-free interval.

A significant negative correlation between lung function in TGF*β*1 in serum but not in induced sputum is an interesting finding. TGF*β*1 can be activated either by an *ανβ*6 or an *ανβ*8-mediated pathway. The *ανβ*6-mediated activation appears to be absolutely dependent on direct cell-cell contact and does not release any diffusible free TGF*β*1 [17]. Such a pathway is ideally suited to the alveolar space. So TGF*β*1 activated by this pathway can have an impact on lung function and will be influenced by high producer genotypes but will not be measureable in BAL, induced sputum, or blood. Release of *free* TGF*β*1 is provided by activation by *ανβ*8 integrin in the conducting airways [18]. This mechanism does not depend on direct cell-cell contact and should provide TGF*β*1 able to diffuse away and to affect cells at a distance. Moreover, *ανβ*8 mRNA is also expressed on a variety of leukocytes [19]. So our protocol was only able to assess the *ανβ*8-dependent part of TGF*β*1 contributing to lung disease. 

Another reason for a significant negative correlation between lung function in TGF*β*1 in serum but not in induced sputum could be attributed to higher stability of TGF*β*1 in blood and its regulation. The circulatory system is characterized by an in and outflow, but the airways are a one-way system. So the influences of confounders like other inflammatory mediators in the lung can be an explanation for the lack of a correlation sputum TGF*β*1 and lung function. 

We could find higher TGF*β*1 levels in serum in CF patients than in the CF cohort of Schwarz and co-workers [8]. The differences between their findings and ours can be explained by the following, we assessed TGF*β*1 in serum and they measured it in plasma of heparinized blood. 

Moreover, Harris et al. [11] also reported higher TGF*β*1 levels in serum than in plasma.

One reason why we were not able to see any difference by genotype might be that we did not compare extreme phenotypes. But most importantly this study was not sufficiently powered to evaluate for significant associations between TGF*β*1 genotype and serum TGF*β*1 levels. Interestingly the two mutants examined occurred together in all but one patient (pt no. 9, excluded from the analysis as he was to young to perform lung function). So, we can speculate about a linkage disequilibrium. Evidence of a linkage disequilibrium (*D*′ = 0.94) for carriers of the C−509T and T869C SNP was provided by Guo et al. [20] for patients with increased risk of gastric cardia adenocarcinoma.

An open question is why Peterson-Carmichael et al. [9] were able to provide evidence of a significant correlation between lung function (FRC) and TGF*β*1 in BAL. First we did not measure FRC; second we cannot exclude that in a cohort of a median age of 86 weeks with 55.6% *P. aeruginosa* colonization the interaction of endotoxin and TGF*β*1 [7] had an impact on lung function. In our cohort (median age 20.5 y), only 36% were colonized with *P. aeruginosa*. The TGF*β*1 in the BAL was higher compared to our patients (median 107 versus 76.5 pg/mL, range (45–354) versus (42.2–195)). In addition, we cannot exclude that the percentage of a high-producer genotype was higher in their cohort. The data from Harris et al. [11] on TGF*β*1 in BAL from older children (median 9.1 y) do not provide evidence that the high-producer genotype has an impact on TGF*β*1 levels as only one out of 25 genotyped patients had the CC codon 10 genotype. Moreover, as BAL samples were collected in children experiencing a respiratory exacerbation, secondary effects of ongoing infections cannot be ruled out. The higher TGF*β*1 levels in their study compared to our cohort might also be attributed to that (135 versus 76.5 pg/mL). Second, as only 68% of their patients were delta F508 homozygous, the groups are not homogenous in terms of their CFTR mutation. 

Different to the study by Harris et al. [11] with an inverse association of *plasma* TGF*β*1 with diminished lung function after antibiotic therapy of a pulmonary exacerbation, we were able to show a significant negative correlation between lung function and TGF*β*1 in *serum* in an exacerbation-free interval. The reason to assess TGF*β*1 in serum and not in plasma was to study a mechanism of disease. We were interested in the propensity to produce TGF*β*1, to prove the high-producer hypothesis. According to the manufacturer's manual, serum serves this purpose better than plasma. As discussed in the second paragraph, the possibility to assess TGF*β*1 *ex vivo* is limited and it cannot be ruled out that the majority of TG*β*1 effects cannot be assessed *ex vivo*. Our aim was not to establish a biomarker, which was one of the results of the intervention-related study of Harris et al. 

The only study which could provide evidence of a correlation between TGF*β*1 in plasma of 56 CF patients and TGF*β*1 genotype is the French-German study by Corvol et al. [4]. Higher plasma levels of TGF*β*1 were observed in patients carrying the TGF*β*1 +869TT genotype (mean value 35.0 ng/mL). The +869CT genotype had an intermediate TGF*β*1 level (22.9 ng/mL) and the +869 CC genotype had lower TGF*β*1 levels (8.7 ng/mL). Interestingly, this genotype as well as the higher producer +869TT level had a higher rate of decline in LFT than the +869CT genotype. Those with the lowest TGF*β*1 levels in plasma had more severe phenotype than those with the CT genotype. Their data might question the value of measurements of TGF*β*1 in plasma. For the TGF*β*1 polymorphism at position −509 there was no significant association with TGF*β*1 plasma levels.

Vital capacity values in this study correlated better with TGF*β*1 in serum than FEV1 and MEF 25%. Concerning the proinflammatory cytokines IL-8 and TNF-alpha, there was no correlation with VC, reflecting the profibrotic effect which is a specific feature of TGF*β*1. 

Interestingly, cytokines in induced sputum as IL-8 and TNF-alpha correlated significantly with TGF*β*1. An explanation might be provided by the following: bacterial flagellin is part of bacteria like *P. aeruginosa* [21], *S. aureus* [22], and *S. maltophilia* [23]. Yang et al. [12] provided evidence that flagellin from *P. aeruginosa* induces TGF*β*1 and IL-8 expression in normal bronchial epithelial cells *in vitro* via MAP kinases. 

In our study there were correlations for TGF*β*1 and IL-8 as well as TNF-alpha in sputum regardless if the patients were positive or negative for *P. aeruginosa*. But if analyzing the correlation by carrier status of bacteria with flagellin (*P. aeruginosa, S. aureus, or S. maltophilia*), the data are more distinctive. For those who were negative, there was no correlation, but for those who had microbiological evidence of at least one bacteria the correlation was positive (TGF*β*1/IL-8 *r* = 0.593, *P* < 0.007; TGF*β*1/TNF alpha *r* = 0.536, *P* < 0.018).

So colonization with bacteria with flagellin (*P. aeruginosa, S. aureus, or S. maltophilia*) might be the reason for coexpression of TGF*β*1 and cytokines like IL-8 or TNF-alpha in the CF lung. This might prompt the clinician to favor eradication of these bacteria.

What is striking are the extremely high IL-8 levels in IS compared to TNF alpha. Explanations are the following: downregulation of IL-8 receptors in the presence of elastase [24], ribonuclear protein hnRNP (A2B1) binding to the IL-8 promoter, and hyperproduction of IL-8 mRNA in bronchial epithelial cells [25]. Moreover, Sagel et al. [26] also found tremendous differences between IL-8 and TNF alpha in IS in a longitudinal cohort study with 35 CF children with annual measurement for a period over 3 years.

Another effect of TGF*β*1 is the increase in alveolar endothelial permeability [7]. We interpret the higher leukocyte levels in sputum of CF patients compared to controls in this regard. This effect is robust even when analyzing only patients who are negative for *P. aeruginosa*.

In conclusion, the findings of this study provide evidence that lung function correlates with TGF*β*1 in serum but not in induced sputum. Genotypes in this small cohort did not have an impact on TGF*β*1 in serum or sputum neither lung function. In IS, there might be a concomitant upregulation of TGF*β*1 and IL-8 as well as TNF alpha in CF patients colonized by bacteria with flagellin like *P. aeruginosa, S. aureus, and S. maltophilia*.

## Figures and Tables

**Table 1:** Patients characteristics.

	CF patients	Controls
	(*n* = 24)	(*n* = 8)
	Median	
ΔF 508 (homozygous)	+	−
Age (years)	20.5	27.5
Sex (m/f)	(14/10)	(3/5)
BMI (kg/m^2^)	20.4	21.6
*P. aeruginosa + *	9	−
Shwachman score	75	−
FEV1 (% predicted)	83	−
MEF 25 (% predicted)	36.7	−
VC (% predicted)	84.9	−

ΔF 508 (homozygous): homozygous for the ΔF 508 mutation; m: male; f: female; *P. aeruginosa +*: *Pseudomonas aeruginosa* colonization; FEV1 (% predicted): forced expiratory volume in 1 s in % predicted; MEF 25 (% predicted): maximum expiratory flow at 25% of forced vital capacity in % predicted; VC (% predicted): vital capacity in % predicted.

**Table 2:** TGF*β*1 and leucocytes in blood and sputum.

	CF patients	Control
	(*n* = 24)	(*n* = 8)
	Median	
TGF*β*1 (pg/mL)		
Serum	35.1 × 10^3^	—
Sputum	76.5*	59.1*
Leucocytes (/*μ*L)		
EDTA blood	6600	6800
Sputum	520^#^	60^#^

*P < 0.074, ^#^ *P* < 0.036.

**Table 3:** IL-8 and TNF-alpha in whole blood and sputum.

	CF patients	Control
	(*n* = 24)	(*n* = 8)
	Median	
IL-8 (pg/mL)		
Whole blood	12*	5*
Sputum	5791*	28.3*
TNF-alpha (pg/mL)		
Whole blood	11.6	17
Sputum	29.6*	16.8*

*P < 0.01.

**Table 4:** TGF*β*1 variant −509C/T. Patients characteristics.

	Mutant	Wildtype
	(*n* = 9)	(*n* = 14)
	Median	
Alter (years)	24	25.5
Sex (m/f)	(4/5)	(9/5)
BMI (kg/m^2^)	21.9	19.2
Shwachman score	75	75
FEV1 (% predicted)	68.6	86.7

**Table 5:** TGF*β*1 variant +869T->C. Patients characteristics.

	Mutant	Wildtype
	(*n* = 9)	(*n* = 14)
	Median	
Alter (years)	24	25.5
Sex (m/f)	(4/5)	(9/5)
BMI (kg/m^2^)	21.9	19.2
Shwachman score	75	75
FEV1 (% predicted)	68.6	86.7

**Table 6:** TGF*β*1 variant −509C/T. TGF*β*1 and leucocytes.

	Mutant	Wildtype
	(*n* = 9)	(*n* = 14)
	Median	
TGF*β*1 (pg/mL)		
Serum	35.8 × 10^3^	34.9 × 10^3^
Sputum	80.2	71.8
Leucocytes (/*μ*L)		
EDTA blood	6325	6990
Sputum	651	495

**Table 7:** TGF*β*1 variant +869T->C. TGF*β*1 and leucocytes.

	Mutant	Wildtype
	(*n* = 9)	(*n* = 14)
	Median	
TGF*β*1 (pg/mL)		
Serum	35.8 × 10^3^	34.9 × 10^3^
Sputum	80.2	71.8
Leucocytes (/*μ*L)		
EDTA blood	6325	6990
Sputum	651	495
